# Cricotracheal Resection for Subglottic Stenosis following Prior Dissection and Tracheostomy

**DOI:** 10.70352/scrj.cr.25-0810

**Published:** 2026-04-21

**Authors:** Kohei Hashimoto, Koichi Hirano, Yoko Nakazato, Akira Motoyasu, Hiroyuki Seki, Kiyoshi Moriyama, Kazuharu Suda, Keisei Tachibana, Ryota Tanaka, Haruhiko Kondo

**Affiliations:** 1 Department of Thoracic and Thyroid Surgery, Kyorin University, School of Medicine, Tokyo, Japan; 2 Department of Anesthesiology, Kyorin University, School of Medicine, Tokyo, Japan

**Keywords:** cricotracheal resection, tracheal stenosis, airway surgery

## Abstract

**INTRODUCTION:**

Cricotracheal resection is a definitive treatment for subglottic tracheal stenosis. This procedure can be challenging in the redo setting.

**CASE PRESENTATION:**

A 32-year-old man was referred to our department with post-intubation subglottic tracheal stenosis related to diabetic ketoacidosis 5 years ago. The stenosis was approximately 80% starting 2 cm below the glottis. A tracheal resection was attempted in the same year, but resulted in a tracheostomy due to strong adhesions around the trachea. The patient was followed up while maintaining spontaneous breathing and phonation. However, he desired stoma closure and was scheduled for reoperation. The patient was intubated in the supine position through the existing tracheostomy, and an incision was made along the previous incision. The adhesions around the trachea were meticulously dissected. On the cephalic side, a longitudinal incision was made through the anterior wall of the scarred trachea while preserving the subglottic mucosa. The trachea was transected at the level of the existing tracheostomy, and the caudal end of the trachea was dissected and resected just below the tracheostomy. The mucosa was further dissected from the cricoid cartilage, and the anterior cricoid arch was resected. The stenosed mucosa was then resected. The posterior wall of the mucosa was sutured continuously with 4-0 polydioxanone, and the anterior wall was anastomosed with interrupted 3-0 polydioxanone under intermittent apnea. Ventilation was resumed with a laryngeal mask. A tracheostomy was placed 1.5 cm below the anastomosis. The patient was discharged on POD 19 without complications. The tracheostomy tube was removed 2 months after surgery, and bronchoscopy 3 months after surgery confirmed a wide patent anastomosis. At 8 months, he has normal phonation and swallowing.

**CONCLUSIONS:**

This redo cricotracheal resection required meticulous dissection and a carefully planned ventilation strategy.

## INTRODUCTION

Even after the advent of low-pressure, high-volume cuffs for endotracheal tubes, modern data suggest that approximately 0.3%–11% of patients develop post-intubation subglottic tracheal stenosis.^1)^ Cricotracheal resection is a definitive treatment for subglottic stenosis, first described by Pearson et al.^2)^ and Grillo^3)^ Successful execution of this procedure requires a detailed understanding of subglottic airway anatomy and meticulous dissection techniques. Redo cases can be especially challenging, as previous interventions may distort normal anatomical planes and increase surgical risk.

## CASE PRESENTATION

A 32-year-old man was referred to our department with post-intubation subglottic tracheal stenosis that developed following an episode of diabetic ketoacidosis requiring oral intubation, rather than tracheostomy, 5 years prior. He recovered from intensive care and was discharged; however, he subsequently experienced progressive exertional dyspnea. Evaluation revealed subglottic tracheal stenosis involving approximately 80% of the airway lumen, beginning 2 cm below the glottis.

**Fig. 1:**
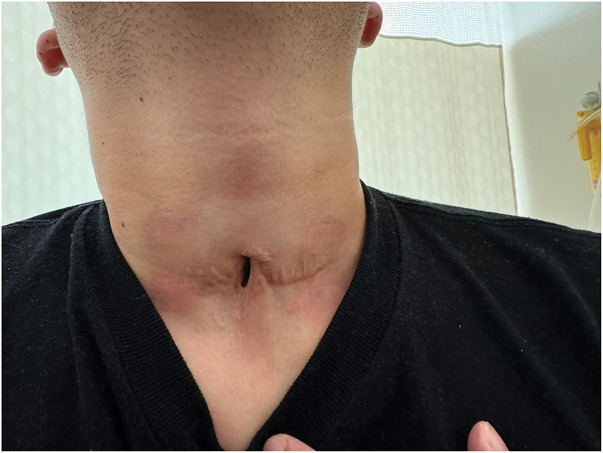
A young male with subglottic tracheal stenosis with a salvage tracheostomy.

An initial attempt at tracheal resection was performed by the head and neck surgery team in the same year. However, due to dense adhesions around the trachea, the procedure was unsuccessful and ultimately resulted in a permanent tracheostomy. The patient was managed conservatively thereafter, maintaining spontaneous breathing and phonation through both the tracheostomy stoma and the vocal cords (**Fig. 1** and **Video 1**). Given the patient’s strong desire for tracheostomy closure, a reoperation was planned. A multidisciplinary team comprising thoracic surgeons, head and neck surgeons, and anesthesiologists was assembled to undertake this complex revision surgery.

The patient was anesthetized and intubated in the supine position via the existing tracheostomy. In the previous surgery, the strap muscles and thyroid were divided in the midline, and the cricothyroid muscle was not dissected. A cervical incision was made along the previous scar, and dense adhesions surrounding the trachea were meticulously dissected. Based on the intraoperative impression of the surgeon who scrubbed in for both procedures, the adhesions were more severe during the redo operation. Special care was taken to stay close to the tracheal wall—essentially shaving along the tracheal surface—during lateral dissection to avoid injury to the recurrent laryngeal nerves. Intraoperative nerve monitoring was not used, as we considered that the safest strategy in this redo setting to avoid recurrent laryngeal nerve injury was to remain strictly on the tracheal wall, rather than attempting to identify the nerves. On the cephalic side, a longitudinal incision was made through the anterior wall of the scarred trachea, preserving the subglottic mucosa (**Video 2**). The trachea was transected at the level of the existing tracheostomy, and the distal (caudal) segment of the trachea (approximately 3 cm in total length) was dissected and resected just below the stoma (**Video 3**). The mucosa was further mobilized from the cricoid cartilage, and the anterior cricoid arch (approximately half of the cricoid circumference) was resected. The stenotic mucosa was then excised.

Tracheal reconstruction was performed by first suturing the posterior wall of the mucosa with a continuous 4-0 polydioxanone suture, followed by anterior wall anastomosis using interrupted 3-0 polydioxanone sutures under intermittent apnea. Ventilation was re-established using a laryngeal mask. A flexible drain was placed, and the neck wound was closed in layers. A new tracheostomy was created 1.5 cm below the anastomosis. A chin stitch was not used (**Video 4**).

The patient was discharged on POD 19 without complications. The tracheostomy tube was successfully removed 2 months after surgery. Follow-up bronchoscopy at 3 months confirmed a widely patent anastomosis. At 8 months, the patient demonstrated normal phonation and swallowing function (**Video 5**). Written informed consent for publication was obtained from the patient; institutional review board approval was not required.

## DISCUSSION

In addition to cricotracheal resection, various treatment modalities for post-intubation subglottic tracheal stenosis have been reported, including topical steroid injection, application of mitomycin C, tracheostomy, stenting, and airway dilatation using balloon, rigid bronchoscopy, or laser techniques. However, these non-resectional approaches are associated with a higher recurrence rate than definitive surgical resection. Cricotracheal resection is therefore considered the gold standard treatment for this condition,^4)^ particularly in patients who are otherwise fit.

Cricotracheal resection may be performed by either head and neck surgeons or thoracic surgeons; however, the procedure lies at the interface of both specialties and requires the combined skill set and knowledge.^5)^ We believe that differences in surgical strategy and team structure may have contributed to the outcome. In particular, the present operation was performed within a multidisciplinary framework involving thoracic surgeons, head and neck specialists, and anesthesiologists, whereas the initial procedure was performed by a more limited team.

A multidisciplinary team approach was also important for the management of potential postoperative swallowing dysfunction and voice impairment related to recurrent laryngeal nerve injury or vocal cord edema. In fact, major airway programs worldwide typically involve close collaboration between thoracic surgeons and head and neck specialists. Reports of redo cricotracheal resection are relatively scarce,^6)^ although its feasibility has been reported. Similar to initial cricotracheal resection, our case highlights that favorable outcomes can be achieved in selected patients through meticulous dissection and appropriate tension management. In this case, a chin stitch was not used. Postoperative neck flexion was carefully maintained with a supportive cradle. Previous reports have also suggested that laryngotracheal resection can be performed safely without a guardian chin stitch.^7)^

In redo cases, previous surgeries often obscure normal anatomical planes, making dissection significantly more challenging and increasing the risk of recurrent laryngeal nerve injury. A well-coordinated ventilation strategy in close collaboration with anesthesiologists is also crucial. These situations require a high level of surgical expertise.

Given these complexities, the involvement of a multidisciplinary team is indispensable—particularly in technically demanding cases such as ours.

## CONCLUSIONS

In conclusion, this redo cricotracheal resection required meticulous dissection and a carefully planned ventilation strategy. The complexity of such airway procedures underscores the importance of a multidisciplinary team approach.

## SUPPLEMENTARY MATERIALS

Videos 1–5 Procedure and pre-/postoperative bronchoscopic findings.

## DECLARATIONS

### Data availability statement

All data are incorporated into the article and its online supplementary material.
